# Failures in Business

**Published:** 1871-04

**Authors:** 


### FAILURES IN BUSINESS.

WHAT untold agonies wrench the heart of an honorable business man, as he sees himself approaching the maelstrom which is to engulf the savings of a life-time, and more than that, which leaves him a debtor to those who once trusted him, to say nothing of the enduring wretchedness of seeing his own loved ones living in self-denial and destitution, hopeless of any remedy from his hands! During 1870, three thousand five hundred men failed in business, owing to other persons one hundred millions of dollars.

To be old and poor, and have no home, is terrible beyond expression; but how many these failures have sent to a premature grave, and, worse, to a mad-house, and, worse still, to crime and bodily degradation, only the Judgment can disclose. What hopes have been blasted, what hearts wilted and withered away, no one may know. But a near question comes home to the reader: "Who knows but I may fail before another year? who knows but that I may be in an early grave, my wife in the mad-house, my son in the penitentiary, and my daughter on the street, as not remote results of such an event, and how may I certainly prevent it?"

Some men fail by trusting others, some by being trusted. The man has never yet been found who was willing to acknowledge that he failed because he had no more sense. It is always somebody else that broke him.

Solomon said, "He that hateth suretyship is sure." Wonder if he found that out by having endorsed somebody's note!

"THE MASTER"

would not trust himself to man, because He knew what was in man, that the human heart was deceitful above all things, and desperately wicked.

The man who trusts, and the man who is trusted, aim at the same result; both hope to make money by the operation, and very often both lose. If all business was done on the basis of a "cash transaction," or, as John Randolph expressed it,

"PAY AS YOU GO,"

human suffering, and sorrow, and sickness, would be diminished by one half, and human crime and human curse would largely disappear. Two short rules might serve to abate much of the mental misery and moral degradation of debt.

Never trust out more than you can afford to lose.

Never engage to pay more than you have money to pay already in your pocket, and the next minute pay it out.

If everybody were to observe these two simple rules, this world would be better and happier in a year than it has been since Eve found out that she wanted a better dress.

